# Supplementary material for: Nucleophilic Substitution at Tetracoordinate Sulfur. Kinetics and Mechanism of the Chloride-Chloride Exchange Reaction in Arenesulfonyl Chlorides: Counterintuitive Acceleration of Substitution at Sulfonyl Sulfur by ortho-Alkyl Groups and Its Origin
Source: Molecules. 2020 Mar 20;25(6):1428. doi: 10.3390/molecules25061428 (PMC7144404; doi:10.3390/molecules25061428)
Supplement: Supplementary file 1 [file molecules-25-01428-s001.pdf]

Supplementary Material for

Kinetics nad Mechanism of the Chloride Exchange Reaction in Arenesulfonyl Chlorides: Counterintuitive Acceleration of Substitution at Sulfonyl Sulfur by *ortho*-Alkyl Groups and its Origin

Marian Mikołajczyk*[a], Monika Gajl[a], Jarosław Błaszczyk[a], Marek Cypryk*[b] and Bartłomiej Gostyński[b]

Centre of Molecular and Macromolecular Studies, Polish Academy of Sciences,
90-363 Lodz, Sienkiewicza 112, Poland

[a] Department of Organic Chemistry

[b] Department of Structural Chemistry

*Corresponding author:

Marian Mikołajczyk, marmikol@cbmm.lodz.pl

Marek Cypryk, mcypryk@cbmm.lodz.pl

List of Contents

Table **S1**. Comparison of selected geometrical parameters for crystal and DFT structures of **(4-Me)-1**

Table **S2**. Comparison of selected geometrical parameters for crystal and DFT structures of **(2,4,6-Me$_3$)-1**

Table **S3**. Comparison of selected geometrical parameters for crystal and DFT structures of **(2,6-*i*Pr$_2$)-1**

Table **S4**. Calculated selected bonding parameters for **1** and **(2,6-Me$_3$)-1**

Table **S5**. Energies of electron delocalization obtained by NBO6 deletion procedure

Table **S6**. B3LYP-GD3/6-31+G(d) calculated electronic energies (E), thermal corrections to enthalpies (ΔH), thermal corrections to Gibbs free energies (ΔG), B3LYP-GD3/6-311+G(2d,p)//B3LYP-GD3/6-31+G(d) electronic energies (E(TZ)), enthalpies (H), and Gibbs free energies (G), in the gas phase and the imaginary frequencies (Im Freq, cm^{-1}) of the transition states.

Table **S7**. B3LYP-GD3/6-31+G(d) calculated electronic energies (E), thermal corrections to enthalpies (ΔH), thermal corrections to Gibbs free energies (ΔG), B3LYP-GD3/6-311+G(2d,p)//B3LYP-GD3/6-31+G(d) electronic energies (E(TZ)), enthalpies (H), and Gibbs free energies (G), in acetonitrile and the imaginary frequencies (Im Freq, cm^{-1}) of the transition states.

Table **S8**. B3LYP-GD3/6-311+G(2d,p) calculated electronic energies (E), thermal corrections to enthalpies (ΔH), thermal corrections to Gibbs free energies (ΔG), enthalpies (H), and Gibbs free energies (G), in the gas phase and the imaginary frequencies (Im Freq, cm^{-1}) of the transition states.

Table **S9**. B3LYP-GD3/6-311+G(2d,p) calculated electronic energies (E), thermal corrections to enthalpies (ΔH), thermal corrections to Gibbs free energies (ΔG), enthalpies (H), and Gibbs free energies (G), in acetonitrile and the imaginary frequencies (Im Freq, cm^{-1}) of the transition states.

Section **S10.** B3LYP-GD3/6-311+G(2d,p) calculated geometries of arenesulfonyl chlorides, RSO_2Cl, reactant complexes, RSO_2Cl-Cl^- (RC), and transition states, $RSO_2Cl_2^-$ (TS)

Table S1. Comparison of selected geometrical parameters for crystal and DFT structures of **(4-Me)-1**, calculated with B3LYP-GD3/6-311+G(2d,p) and APFD/6-311+G(2d,p) methods

**Bonding parameters**	**Calculated (gas)**	**Calculated (MeCN)**	**Crystal structure**
Bond lengths [Å]	B3LYP (APFD)	B3LYP (APFD)	
r(S1-Cl1)	2.132 (2.092)	2.141 (2.095)	2.046
r(S1-C1)	1.774 (1.759)	1.764 (1.750)	1.745
r(S1-O1), r(S1-O2)	1.440 (1.433)	1.443 (1.437)	1.420, 1.416
Angles [°]			
α(O1-S1-O2)	122.3 (122.2)	121.1 (121.0)	120.6
α(C1-S1-Cl1)	100.6 (100.8)	101.7 (101.8)	101.3
Dihedral angles [°]			
θ(C2-C1-S1-O1)	-21.4 (-21.3)	-21.6 (-21.6)	-15.7
θ(C6-C1-S1-O2)	21.4 (21.3)	21.6 (21.6)	28.8
θ(C6-C1-S1-Cl1)	-89.9 (-90.1)	-89.7 (-89.9)	-82.9

Table S2. Comparison of selected geometrical parameters for crystal and DFT structures of **(2,4,6-Me$_3$)-1**, calculated with B3LYP-GD3/6-311+G(2d,p) and APFD/6-311+G(2d,p) methods

**Bonding parameters**	**Calculated (gas)**	**Calculated (MeCN)**	**Crystal structure**
Bond lengths [Å]	B3LYP (APFD)	B3LYP (APFD)	
r(S1-Cl1)	2.147 (2.104)	2.159 (2.110)	2.037
r(S1-C1)	1.791 (1.772)	1.780 (1.762)	1.766
r(S1-O1), r(S1-O2)	1.439 (1.433)	1.443 (1.436)	1.409, 1.412
Angles [°]			
α(O1-S1-O2)	120.1 (120.0)	119.2 (119.0)	117.8
α(C1-S1-Cl1)	100.4 (100.5)	101.4 (101.5)	101.6
Dihedral angles [°]			
θ(C2-C1-S1-O1)	-13.7 (-22.2)	-11.3 (-16.8)	-17.6
θ(C6-C1-S1-O2)	30.3 (22.2)	32.6 (27.7)	28.9
θ(C6-C1-S1-Cl1)	-81.1 (-89.3)	-78.6 (-83.8)	-83.9

Table S3. Comparison of selected geometrical parameters for crystal and DFT structures of **(2,4,6-*i*Pr$_3$)-1**, calculated with B3LYP-GD3/6-311+G(2d,p)//B3LYP/6-31+g(d) method (see Fig. S1 for description of atom numbering).

**Bonding parameters**	**Calculated (gas)**	**Calculated (MeCN)**	**Crystal structure**
Bond lengths [Å]			
r(S-Cl)	2.156	2.167	2.058
r(S-C5)	1.810	1.801	1.776
r(S-O3), r(S-O4)	1.460, 1.459	1.464, 1.462	1.424
r(H34-O3), r(H14-O4)	2.281, 2.198	2.303, 2.197	2.283, 2.159
Angles [°]			
α(O3-S-O4)	119.0	118.0	118.1
α(C5-S-Cl)	101.9	103.1	102.4
α(C9-C5-S)	170.2	170.0	172.4
α(C33-H34-O3), α(C13-H14-O4)	113.4, 116.4	113.6, 117.3	119.4, 122.2
Dihedral angles [°]			
θ(C6-C5-S-O3)	29.1	31.2	35.7
θ(C12-C5-S-O4)	-13.2	-10.9	-6.3
θ(C6-C5-S-Cl)	-83.4	-81.3	-77.6

[a]) Crystal structure of 2,4,6-triisopropylbenzenesulfonyl chloride, **(2,4,6-*i*Pr$_3$)-1** [ref. 29]

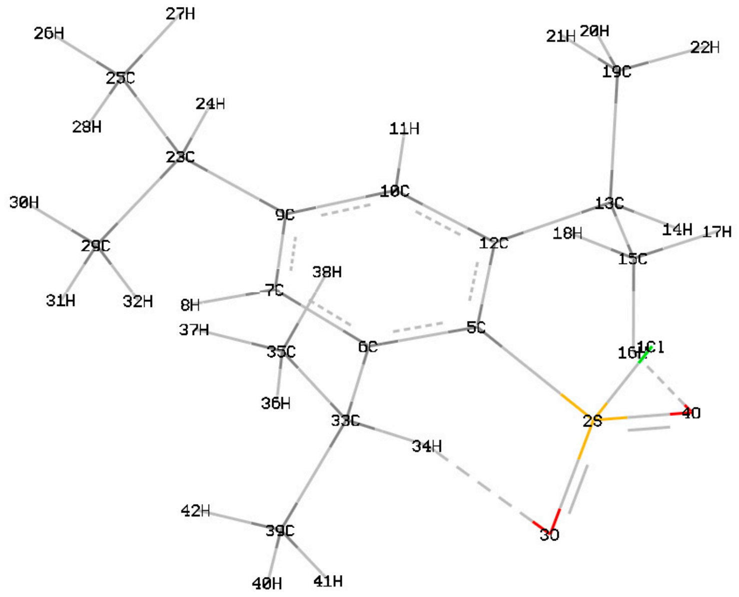

Fig. S1. **(2,4,6-*i*Pr$_3$)-1,** atom numbering; weak hydrogen bonds marked by dashed lines.

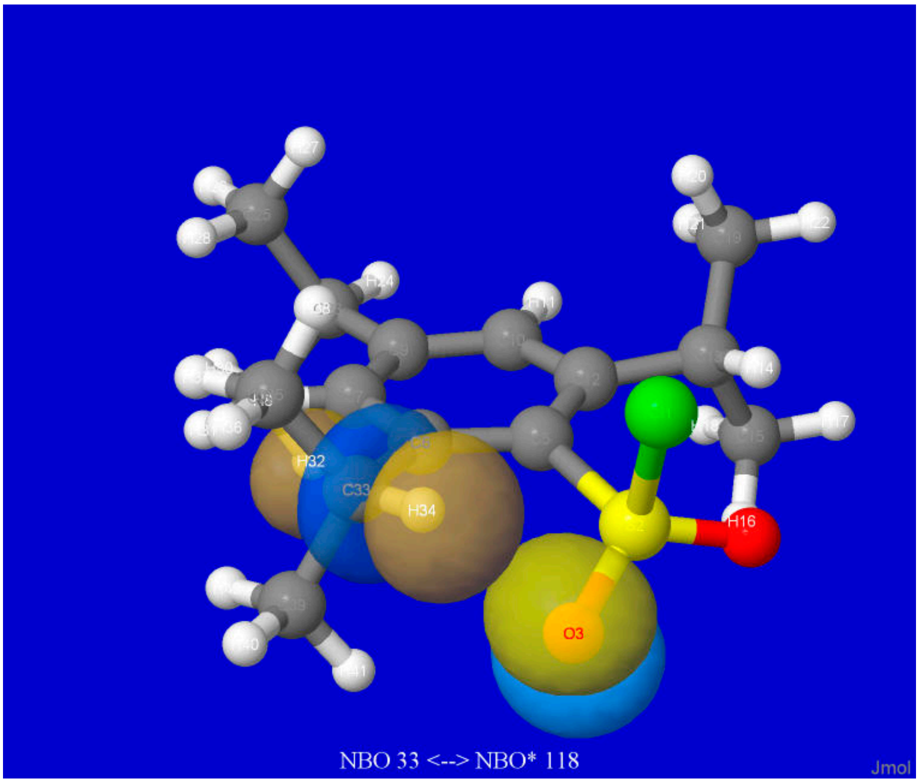

Fig. S2. Visualisation of the delocalization interaction between O3 and C33-H34 ($n_{O} \rightarrow \sigma^{*}_{H\text{-}C}$) of *ortho*-isopropyl group according to NBO analysis. This Figure was created using Jmol [Jmol: an open-source Java viewer for chemical structures in 3D. http://www.jmol.org] and Jmol-NBO 2.1 [script generation: M. Patek, http://chemgplus.blogspot.com/2013/08/jmol-nbo-visualization-helper.html].

Table S4. Comparison of selected bonding parameters for **1** and **(2,6-Me$_2$)-1**, calculated using B3LYP-GD3/6-311+G(2d,p) and APFD/6-311+G(2d,p) methods

**Bonding**	**1 (gas)**	**1 (MeCN)**	**(2,6-Me$_2$)-1**	**(2,6-Me$_2$)-1**
Bond lengths [Å]	B3LYP (APFD)	B3LYP (APFD)	B3LYP (APFD)	B3LYP (APFD)
r(S1-Cl1)	2.129 (2.089)	2.134 (2.092)	2.143 (2.101)	2.154 (2.106)
r(S1-C1)	1.780 (1.764)	1.772 (1.756)	1.798 (1.778)	1.789 (1.770)
r(S1-O1), r(S1-O2)	1.440 (1.433)	1.443 (1.437)	1.438 (1.432)	1.442 (1.435)
Angles [°]				
α(O1-S1-O2)	122.4 (122.3)	121.2 (121.1)	120.1 (120.0)	119.2 (119.1)
α(C1-S1-Cl1)	100.4 (100.6)	101.4 (101.5)	100.1 (100.3)	101.1 (101.2)
Dihedral angles [°]				
θ(C2-C1-S1-O1)	21.2 (21.2)	21.5 (21.6)	14.0 (16.7)	10.9 (14.7)
θ(C6-C1-S1-O2)	-21.2 (-21.2)	-21.5 (-21.6)	-29.9 (-27.2)	-32.6 (-29.4)
θ(C6-C1-S1-Cl1)	90.1 (90.2)	90.0 (90.0)	81.7 (84.4)	78.7 (82.2)

Table S5. E(2) energy values for major donor-acceptor interactions in arenesulfonyl chlorides obtained by NBO6 deletion procedure (kcal/mol)

**Comp.**	**SO$_2$Cl↔ring**	**n_O→ σ^*_{S-C}**	**n_O→ σ^*_{S-O}**	**n_O→ σ^*_{S-Cl}**	**n_O→d_S**	**n_{Cl}→d_S**	**n_{Cl}→ σ^*_{S-O}**	**n_{Cl}→ σ^*_{S-C}**
**1**	32.0	31.8	51.4	57.4	96.1	6.0	7.5	1.5
**1** (rot)	31.7							1.9
**(4-Me)-1**	33.7	31.6	51.4	57.3	95.7	5.9	7.4	1.5
**(2,6-Me$_2$)-1**	38.5	30.9	50.1	58.4	95.6	5.6	7.3	1.3
**(2,4,6-Me$_3$)-1**	36.4	31.3	50.1	58.4	96.0	5.6	7.3	1.4
**(2,4,6-Me$_3$)-1** (rot)	37.1							

SO$_2$Cl↔ring means the sum of interactions between two molecular units, mainly n_{Cl}→π^*_{CC} and n_O→π^*_{CC} delocalizations

d_S means RY(1)-RY(5) Rydberg orbitals on sulfur, according to NBO6, thus, n_X→d_S correspond to the sum of delocalizations from lone pairs of X to RY(1)-RY(5) of S;

(rot) means that the SO$_2$Cl group was rotated by 10° from minimum energy geometry

Table **S6**. B3LYP-GD3/6-31+G(d) calculated electronic energies (E), thermal corrections to enthalpies (ΔH), thermal corrections to Gibbs free energies (ΔG), B3LYP-GD3/6-311+G(2d,p)//B3LYP-GD3/6-31+G(d) electronic energies (E(TZ)), enthalpies (H), and Gibbs free energies (G), in the gas phase and the imaginary frequencies (Im Freq, cm^{-1}) of the transition states. All energies are given in hartrees

**Structure**	**E (1)**	**ΔH (2)**	**ΔG (3)**	**E(TZ) (4)**	**H (4)+(2)**	**G (4)+(3)**	**Im Freq**
Sulfonyl chlorides							
**1**	-1240.44761	0.10966	0.06422	-1240.63477	-1240.57055	-1240.52511	
**1···Cl$^-$(RC)**	-1700.73684	0.11177	0.05928	-1700.95374	-1700.89446	-1700.84197	
**1-Cl$^-$(TS)**	-1700.73454	0.11050	0.06003	-1700.95252	-1700.89249	-1700.84202	-112.08
**(4-Me)-1**	-1279.77027	0.13848	0.08765	-1279.96772	-1279.88007	-1279.82924	
**(4-Me)-1-Cl$^-$(RC)**	-1740.05806	0.14061	0.08263	-1740.28511	-1740.20248	-1740.14450	
**[(4-Me)-1-Cl]$^-$(TS)**	-1740.05531	0.13929	0.08330	-1740.28348	-1740.20018	-1740.14419	-117.45
**(2,6-Me$_2$)-1**	-1319.08551	0.16775	0.11632	-1319.29284	-1319.17651	-1319.12509	
**(2,6-Me$_2$)-1···Cl$^-$(RC)**	-1779.37924	0.16992	0.11248	-1779.61623	-1779.50375	-1779.44631	
**[(2,6-Me$_2$)-1-Cl]$^-$(TS)**	-1779.37678	0.16878	0.11432	-1779.61454	-1779.50022	-1779.44577	-120.14
**(2,4,6-Me$_3$)-1**	-1358.40843	0.19663	0.14039	-1358.62599	-1358.48560	-1358.42937	
**(2,4,6-Me$_3$)-1···Cl$^-$(RC)**	-1818.70082	0.19882	0.13674	-1818.94795	-1818.81121	-1818.74913	
**[(2,4,6-Me$_3$)-1-Cl]$^-$(TS)**	-1818.69795	0.19757	0.13776	-1818.94583	-1818.80807	-1818.74826	-125.31
**(2,6-*i*Pr$_2$)-1**	-1476.35142	0.28507	0.22081	-1476.60041	-1476.37960	-1476.31534	
**(2,6-*i*Pr$_2$)-1···Cl$^-$(RC)**	-1936.64814	0.28712	0.21809	-1936.92681	-1936.70872	-1936.63969	
**[(2,6-*i*Pr$_2$)-1-Cl]$^-$(TS)**	-1936.64501	0.28604	0.21882	-1936.92442	-1936.70561	-1936.63839	-115.16
**F$_5$-1**	-1736.59661	0.07379	0.01770	-1736.93394	-1736.91624	-1736.86015	
**F$_5$-1···Cl$^-$(RC)**	-2196.90452	0.07616	0.01309	-2197.27029	-2197.25720	-2197.19413	
**[F$_5$-1-Cl]$^-$(TS)**	-2196.90034	0.07483	0.01333	-2197.26804	-2197.25471	-2197.19321	-131.84
Sulfonyl fluorides							
**1-F**	-880.09989	0.11059	0.06666	-880.28440	-880.21773	-880.17381	
**1-F$_2^-$(RC)**	-980.01404	0.11257	0.06518	-980.22730	-980.16212	-980.11473	
**F$_5$-1-F$^-$**	-1376.24975	0.07472	0.02022	-1376.58455	-1376.56433	-1376.50983	
**F$_5$-1-F$_2^-$ (RC)**	-1476.18686	0.07698	0.02029	-1476.54885	-1476.52856	-1476.47187	

Table **S7**. B3LYP-GD3/6-31+G(d) calculated electronic energies (E), thermal corrections to enthalpies (ΔH), thermal corrections to Gibbs free energies (ΔG), B3LYP-GD3/6-311+G(2d,p)//B3LYP-GD3/6-31+G(d) electronic energies (E(TZ)), enthalpies (H), and Gibbs free energies (G), in acetonitrile and the imaginary frequencies (Im Freq, cm^{-1}) of the transition states. All energies are given in hartrees

**Structure**	**E (1)**	**ΔH (2)**	**ΔG (3)**	**E(TZ) (4)**	**H (4)+(2)**	**G (4)+(3)**	**Im Freq**
Sulfonyl chlorides							
**1**	-1240.45670	0.10939	0.06374	-1240.64299	-1240.57925	-1240.53360	
**1···Cl$^-$(RC)**	-1700.84095	0.11221	0.05779	-1701.05752	-1700.99973	-1700.94531	
**1-Cl$^-$(TS)**	-1700.82287	0.11067	0.05966	-1701.03920	-1700.97954	-1700.92853	-261.44
**(4-Me)-1**	-1279.77972	0.13816	0.08772	-1279.97636	-1279.88863	-1279.83820	
**(4-Me)-1-Cl$^-$(RC)**	-1740.16407	0.14099	0.08089	-1740.39094	-1740.31005	-1740.24995	
**[(4-Me)-1-Cl]$^-$(TS)**	-1740.14553	0.13943	0.08316	-1740.37205	-1740.28889	-1740.23263	-265.3
**(2,6-Me$_2$)-1**	-1319.09335	0.16739	0.11600	-1319.29991	-1319.18391	-1319.13251	
**(2,6-Me$_2$)-1···Cl$^-$(RC)**	-1779.47813	0.17029	0.11044	-1779.71496	-1779.60452	-1779.54468	
**[(2,6-Me$_2$)-1-Cl]$^-$(TS)**	-1779.46260	0.16885	0.11340	-1779.69860	-1779.58520	-1779.52976	-248.41
**(2,4,6-Me$_3$)-1**	-1358.41661	0.19619	0.13986	-1358.63346	-1358.49359	-1358.43727	
**(2,4,6-Me$_3$)-1···Cl$^-$(RC)**	-1818.80129	0.19901	0.13482	-1819.04840	-1818.91357	-1818.84938	
**[(2,4,6-Me$_3$)-1-Cl]$^-$(TS)**	-1818.78563	0.19760	0.13680	-1819.03191	-1818.89510	-1818.83430	-246.13
**(2,6-*i*Pr$_2$)-1**	-1476.35881	0.28460	0.22060	-1476.60712	-1476.38653	-1476.32252	
**(2,6-*i*Pr$_2$)-1···Cl$^-$(RC)**	-1936.74349	0.28760	0.21523	-1937.02203	-1936.80680	-1936.73443	
**[(2,6-*i*Pr$_2$)-1-Cl]$^-$(TS)**	-1936.72797	0.28607	0.21761	-1937.00552	-1936.78790	-1936.71945	-241.5
**(2,4,6-*i*Pr$_3$)-1**	-1594.31834	0.37199	0.29745	-1594.59763	-1594.30019	-1594.22565	
**[(2,4,6-*i*Pr$_3$)-1-Cl]$^-$(TS)**	-2054.68717	0.37347	0.29478	-2054.99582	-2054.70104	-2054.62235	-242.73
**F$_5$-1**	-1736.60619	0.07346	0.01739	-1736.94227	-1736.92488	-1736.86881	
**F$_5$-1···Cl$^-$(RC)**	-2196.99313	0.07629	0.01286	-2197.35948	-2197.34662	-2197.28318	
**[F$_5$-1-Cl]$^-$(TS)**	-2196.97479	0.07479	0.01406	-2197.34116	-2197.32710	-2197.26637	-262.8
Sulfonyl fluorides							
**1-F**	-880.09702	0.11022	0.06595	-880.29280	-880.22685	-880.18259	
**1-F$_2^-$(RC)**	-980.10830	0.11257	0.06513	-980.32065	-980.25552	-980.20809	
**F$_5$-1-F$^-$**	1376.25951	0.07438	0.01984	-1376.59298	-1376.57314	-1376.51860	
**F$_5$-1-F$_2^-$ (RC)**	-1476.26843	0.07676	0.01989	-1476.62971	-1476.60982	-1476.55295	

Table **S8**. B3LYP-GD3/6-311+G(2d,p) calculated electronic energies (E), thermal corrections to enthalpies (ΔH), thermal corrections to Gibbs free energies (ΔG), enthalpies (H), and Gibbs free energies (G), in gas phase and the imaginary frequencies (Im Freq, cm^{-1}) of the transition states. All energies are given in hartrees

**Structure**	**E (1)**	**ΔH (2)**	**ΔG (3)**	**H (1)+(2)**	**G (1)+(3)**	**Im Freq**
Sulfonyl chlorides						
**1**	-1240.63596	0.10944	0.06415	-1240.52652	-1240.57181	
**1···Cl$^-$(RC)**	-1700.95511	0.11148	0.05943	-1700.84363	-1700.89568	
**1-Cl$^-$(TS)**	-1700.95405	0.11040	0.06089	-1700.84365	-1700.89316	-71.1
**(4-Me)-1**	-1279.96894	0.13803	0.08742	-1279.83091	-1279.88152	
**(4-Me)-1-Cl$^-$(RC)**	-1740.28645	0.14013	0.08259	-1740.14632	-1740.20386	
**[(4-Me)-1-Cl]$^-$(TS)**	-1740.28503	0.13903	0.08446	-1740.14600	-1740.20057	-74.6
**(2,6-Me$_2$)-1**	-1319.29409	0.16701	0.11573	-1319.12708	-1319.17836	
**(2,6-Me$_2$)-1···Cl$^-$(RC)**	-1779.61762	0.16914	0.11197	-1779.44849	-1779.50565	
**[(2,6-Me$_2$)-1-Cl]$^-$(TS)**	-1779.61612	0.16803	0.11357	-1779.44809	-1779.50255	-90.69
**(2,4,6-Me$_3$)-1**	-1358.62725	0.19561	0.13949	-1358.43164	-1358.48776	
**(2,4,6-Me$_3$)-1···Cl$^-$(RC)**	-1818.94935	0.19777	0.13565	-1818.75158	-1818.81370	
**[(2,4,6-Me$_3$)-1-Cl]$^-$(TS)**	-1818.94754	0.19662	0.13664	-1818.75093	-1818.81091	-95.69
**(2,6-*i*Pr$_2$)-1**	-1476.60175	0.28353	0.21829	-1476.31822	-1476.38346	
**(2,6-*i*Pr$_2$)-1···Cl$^-$(RC)**	-1936.92821	0.28560	0.21672	-1936.64261	-1936.71149	
**[(2,6-*i*Pr$_2$)-1-Cl]$^-$(TS)**	-1936.92614	0.28458	0.21822	-1936.64156	-1936.70792	-87.22
**F$_5$-1**	-1736.93521	0.07415	0.01843	-1736.86106	-1736.91678	
**[F$_5$-1-Cl]$^-$(TS)**	-2197.26960	0.07534	0.01637	-2197.19427	-2197.25323	-74.22
Sulfonyl fluorides						
**F$_5$-1-F$_2^-$ (RC)**	-1476.55020	0.07724	0.02012	-1476.47296	-1476.53008	

Table **S9**. B3LYP-GD3/6-311+G(2d,p) calculated electronic energies (E), thermal corrections to enthalpies (ΔH), thermal corrections to Gibbs free energies (ΔG), enthalpies (H), and Gibbs free energies (G), in acetonitrile and the imaginary frequencies (Im Freq, cm^{-1}) of the transition states. All energies are given in hartrees

**Structure**	**E (1)**	**ΔH (2)**	**ΔG (3)**	**H (1)+(2)**	**G (1)+(3)**	**Im Freq**
Sulfonyl chlorides						
**1**	-1240.64419	0.10921	0.06381	-1240.53498	-1240.58038	
**1···Cl^-(RC)**	-1701.05874	0.11204	0.05808	-1700.94671	-1701.00067	
**1-Cl^-(TS)**	-1701.04071	0.11057	0.06037	-1700.93014	-1700.98034	-254.43
**(4-Me)-1**	-1279.97758	0.13772	0.08677	-1279.83986	-1279.89081	
**(4-Me)-1-Cl^-(RC)**	-1740.39211	0.14057	0.08140	-1740.25155	-1740.31072	
**[(4-Me)-1-Cl]$^-$(TS)**	-1740.37358	0.13917	0.08370	-1740.23442	-1740.28989	-258.05
**(2,6-Me_2)-1**	-1319.30116	0.16671	0.11558	-1319.13445	-1319.18558	
**(2,6-Me_2)-1···Cl^-(RC)**	-1779.71622	0.16954	0.11010	-1779.54667	-1779.60612	
**[(2,6-Me_2)-1-Cl]$^-$(TS)**	-1779.70024	0.16818	0.11297	-1779.53206	-1779.58727	-249.5
**(2,4,6-Me_3)-1**	-1358.63473	0.19523	0.13906	-1358.43950	-1358.49567	
**(2,4,6-Me_3)-1···Cl^-(RC)**	-1819.04975	0.19810	0.13420	-1818.85165	-1818.91555	
**[(2,4,6-Me_3)-1-Cl]$^-$(TS)**	-1819.03339	0.19673	0.13590	-1818.83666	-1818.89749	-251.12
**(2,6-*i*Pr_2)-1**	-1476.608468	0.28308	0.218785	-1476.32538	-1476.38968	
**(2,6-*i*Pr_2)-1···Cl^-(RC)**	-1937.023312	0.2861	0.214217	-1936.73721	-1936.80910	
**[(2,6-*i*Pr_2)-1-Cl]$^-$(TS)**	-1937.007231	0.28461	0.217026	-1936.72262	-1936.79020	-246.02
**(2,4,6-*i*Pr_3)-1**	-1594.59905	0.36985	0.29467	-1594.22920	-1594.30438	
**(2,4,6-*i*Pr_3)-1···Cl^-(RC)**	-2055.01399	0.37287	0.29131	-2054.64113	-2054.72268	
**[(2,4,6-*i*Pr_3)-1-Cl]$^-$(TS)**	-2054.99748	0.37141	0.29343	-2054.62607	-2054.70405	-247.94
**F_5-1**	-1736.94354	0.07379	0.01807	-1736.86975	-1736.92547	
**F_5-1···Cl^-(RC)**	-2197.36074	0.07662	0.01282	-2197.28412	-2197.34793	
**[F_5-1-Cl]$^-$(TS)**	-2197.3429	0.07522	0.0151	-2197.26768	-2197.32781	-248.51
Sulfonyl fluorides						
**1-F**	-880.294122	0.11006	0.066165	-880.18406	-880.22796	
**1-F_2^-(RC)**	-980.32187	0.11226	0.064711	-980.20961	-980.25716	
**F_5-1-F^-**	-1376.59448	0.07471	0.020541	-1376.51977	-1376.57394	
**F_5-1-F_2^- (RC)**	-1476.63106	0.07703	0.020094	-1476.55404	-1476.61097	

S10. B3LYP-GD3/6-311+G(2d, p) calculated geometries (in Cartesian coordinates) of arenesulfonyl chlorides, RSO_2Cl, reactant complexes, $RSO_2Cl\text{-}Cl^-$, and transition states, $RSO_2Cl_2^-$

$PhSO_2Cl$ (1) (gas)

S 1.2197782099, 0.0000231828, -0.5333777328
O 1.6132546324, -1.2611711986, -1.1051037857
O 1.6132510627, 1.2612619387, -1.1050083225
Cl 1.9710253503, -0.0000516307, 1.4581484678
C -0.5317752725, 0.0000087644, -0.2156643133
C -1.1893846748, 1.2185355974, -0.0981768904
C -1.1893785937, -1.2185289634, -0.098254752
C -2.557724488, 1.2092750181, 0.1411148229
H -0.6405941031, 2.1446052365, -0.2027030828
C -2.5577182677, -1.2092905275, 0.1410378463
H -0.6405835908, -2.144589288, -0.2028402597
C -3.2376169598, -0.0000132942, 0.2626462238
H -3.0922077927, 2.1465821768, 0.2306854008
H -3.0921969547, -2.1466060046, 0.2305488172
H -4.3044975575, -0.0000220077, 0.4500975605

$PhSO_2Cl\text{-}Cl^-$, ($1\cdots Cl^-$(RC)) (gas)

S -1.246105769, 0.3182025551, 0.0000138218
Cl -2.1326290511, -1.7631130718, -0.0000922899
Cl 0.6671643793, 2.7248817035, -0.0000124904
O -1.6908601421, 0.802391392, -1.2767820676
O -1.6908988671, 0.8022735297, 1.276840628
C 0.4389808373, -0.2657082832, 0.0000057632
C 1.0460482933, -0.5366095132, -1.2165811872
C 1.0460596518, -0.536602437, 1.216588575
C 2.3238379137, -1.0773557108, -1.2070921634
H 0.537388449, -0.2940064939, -2.1385668126
C 2.3238487064, -1.0773493905, 1.2070904562
H 0.5374087975, -0.2939936427, 2.1385776263
C 2.9646562251, -1.3473927521, -0.0000030888
H 2.825951409, -1.2759492168, -2.1466134423
H 2.8259707386, -1.275937847, 2.146608213
H 3.9659224283, -1.7627138212, -0.0000065413

$PhSO_2Cl_2^-$, ($1\text{-}Cl^-$(TS)) (gas)

S 1.2233832508, -0.0000018563, -0.0000037054
Cl 0.9322990511, -2.4967787933, 0.000054126
Cl 0.9323086663, 2.4967751068, -0.000060698
O 1.8565320459, 0.0000262586, 1.2939212253
O 1.8565262646, -0.0000329943, -1.2939314709
C -0.5696248204, 0.0000010052, -0.0000009095

C -1.2321558223, 0.0000274137, 1.2155037074
C -1.2321596568, -0.0000230583, -1.2155034154
C -2.6208110545, 0.0000296128, 1.2063979704
H -0.6675251852, 0.0000454152, 2.1371758908
C -2.6208148997, -0.0000204556, -1.206393288
H -0.6675319327, -0.0000430819, -2.1371773745
C -3.3173105727, 0.0000057826, 0.0000034244
H -3.1600469309, 0.0000499288, 2.1465878258
H -3.1600537249, -0.000038928, -2.1465814522
H -4.4013406787, 0.0000076439, 0.0000051438

PhSO2Cl (1) (MeCN)

S 1.2145361057, 0.0000098094, -0.5266072373
O 1.6285481263, -1.2569821245, -1.1015176081
O 1.6285462395, 1.2570199731, -1.10147903
Cl 1.9950608252, -0.0000200532, 1.459863447
C -0.5296352088, 0.0000036533, -0.2159923116
C -1.1846519217, 1.2215852922, -0.0983980682
C -1.1846493904, -1.2215827709, -0.0984328707
C -2.5534829604, 1.21068348, 0.136321081
H -0.6405569493, 2.1507260755, -0.1960410084
C -2.5534803875, -1.2106905077, 0.1362866354
H -0.6405524764, -2.1507196184, -0.1961023938
C -3.2330072307, -0.0000058817, 0.2547935781
H -3.0878975466, 2.1475527564, 0.2251025123
H -3.0878930475, -2.1475634147, 0.2250414045
H -4.3000481776, -0.0000096686, 0.4389328697

PhSO2Cl-Cl$^-$, (1···Cl$^-$(RC)) (MeCN)

S 1.3168549858, -0.0172399285, -0.0000100226
Cl 2.1684548978, -1.9853332415, 0.0000098566
Cl -0.7534499889, 3.5181043807, -0.0000266731
O 1.7156855818, 0.5642182101, 1.2581038693
O 1.7156884155, 0.5641941981, -1.2581341306
C -0.4120026774, -0.3996571152, -0.0000083412
C -1.0609539791, -0.5452257674, 1.2211151028
C -1.0609540401, -0.5452369162, -1.2211303678
C -2.4188145215, -0.8355762487, 1.2105303601
H -0.5216690873, -0.4225136175, 2.150038934
C -2.4188146855, -0.8355871701, -1.2105428206
H -0.5216691391, -0.4225333293, -2.1500553072
C -3.0933374246, -0.9807794914, -0.000005566
H -2.9492142061, -0.9451342068, 2.1475376151
H -2.9492144779, -0.9451535047, -2.147549036
H -4.1523696533, -1.2067272516, -0.0000044728

$PhSO_2Cl_2^-$, (1-Cl^-(TS)) (MeCN)

S 1.1953108075, -0.0000019596, -0.0000010504
Cl 1.0075739859, -2.5722160042, 0.0000756869
Cl 1.0075834212, 2.5722121233, -0.0000825332
O 1.8386471031, 0.0000361194, 1.2868695483
O 1.8386425037, -0.0000428176, -1.2868737462
C -0.582598622, 0.0000009785, -0.0000000568
C -1.2382867894, 0.000021842, 1.2220282804
C -1.2382880987, -0.0000175427, -1.2220276553
C -2.6272788145, 0.0000235734, 1.2098612458
H -0.6831479853, 0.0000346213, 2.149479445
C -2.6272801729, -0.0000143508, -1.2098590448
H -0.6831503227, -0.0000323028, -2.1494794145
C -3.31912811, 0.0000058336, 0.0000014569
H -3.1675326717, 0.0000384006, 2.1478346919
H -3.1675350454, -0.0000272622, -2.1478319073
H -4.4018571886, 0.0000077514, 0.0000020533

4-$MePhSO_2Cl$ ((4-Me)-1) (gas)

S -1.6516331657, 0.0008270552, -0.5044494798
O -2.0715909138, 1.2624275347, -1.0569791459
O -2.0721085087, -1.2602566599, -1.0577654527
Cl -2.3227968702, 0.0003256838, 1.5195202137
C 0.1067771593, 0.0003881829, -0.2664131587
C 0.7748444036, -1.2144522937, -0.1747302188
C 0.7753779519, 1.2148723119, -0.1742294875
C 2.1498842328, -1.2026965359, 0.0070616137
H 0.2265853346, -2.1437009058, -0.2514880298
C 2.1504383141, 1.2024342771, 0.007558682
H 0.227534346, 2.1443981009, -0.2505953725
C 2.8574004317, -0.0002978015, 0.100815183
H 2.6823926815, -2.1439863288, 0.077874252
H 2.6833483131, 2.1434631588, 0.0787579171
C 4.3532936555, -0.0007816588, 0.2678257513
H 4.6940800482, -0.8845839021, 0.8093027021
H 4.8463648391, -0.0033827592, -0.7091681903
H 4.6951907472, 0.8850475403, 0.8052422212

4-$MePhSO_2Cl$-Cl^-, ($[(4\text{-Me})\text{-}1\text{-Cl}]^-$ (RC)) (gas)

S -1.6417723192, 0.0775409635, 0.0000029358
Cl -2.0984997521, -2.1285177923, 0.0000130497
Cl -0.1893306379, 2.9019306397, -0.0000146696
O -2.1691172254, 0.4759012262, -1.2747029413
O -2.1691141714, 0.475912251, 1.2747066703

C 0.1234837666, -0.1288077519, 0.0000019304
C 0.7812189976, -0.2533096407, -1.2130510838
C 0.7812197546, -0.2533124891, 1.2130538773
C 2.1483994094, -0.4817658367, -1.2007924397
H 0.2352883678, -0.1360707216, -2.1382150096
C 2.1484006231, -0.4817683987, 1.2007937465
H 0.2352898755, -0.1360768465, 2.1382187001
C 2.8534431039, -0.592859735, 0.0000004022
H 2.6802128141, -0.5586468592, -2.1430673052
H 2.6802145727, -0.5586518484, 2.1430680711
C 4.3490050641, -0.7809938133, -0.0000017236
H 4.683566784, -1.3280848766, -0.8845630614
H 4.8602903484, 0.1873599848, -0.0000311435
H 4.683575624, -1.3280364553, 0.8845859943

4-MePhSO$_2$Cl$_2^-$, ([(4-Me)-1-Cl]$^-$ (TS)) (gas)
S -1.5720715417, 0.0143584753, 0.0034305025
Cl -1.2996780267, -2.487840879, 0.1972481707
Cl -1.2766323326, 2.5035031013, -0.1902254418
O -2.2065599953, -0.0854457397, -1.2856999034
O -2.2026426352, 0.1148317935, 1.2944300692
C 0.2172992597, 0.0013307005, 0.0017225537
C 0.8828586754, -0.0993033528, -1.2068126282
C 0.8865316153, 0.088636844, 1.2092676416
C 2.2704929047, -0.1087794716, -1.1961207602
H 0.3216745189, -0.1720874511, -2.1277872941
C 2.2741277238, 0.0772912331, 1.1959799781
H 0.3281510005, 0.1593102446, 2.1321090241
C 2.9874679374, -0.018169795, -0.0009692091
H 2.8056203029, -0.1908067654, -2.1364273161
H 2.8121098142, 0.1415352374, 2.1360414275
C 4.4956360336, 0.0093831424, -0.0053784016
H 4.9049689065, -0.4167065476, 0.913599616
H 4.9022908422, -0.5536451809, -0.8487337688
H 4.8699369964, 1.035675411, -0.0856892602

4-MePhSO$_2$Cl ((4-Me)-1) (MeCN)
S -1.6347269217, 0.0008420449, -0.5050678625
O -2.07585812, 1.2581529984, -1.0599434205
O -2.0764799669, -1.2560692914, -1.0603546629
Cl -2.3451623347, 0.0006856297, 1.5142361214
C 0.1134684348, 0.0003692327, -0.2680186654
C 0.7794054909, -1.2177241216, -0.1723899195
C 0.780000324, 1.2180897837, -0.1719712194
C 2.1533475006, -1.2046025119, 0.0105891997

H 0.236548421, -2.1500692252, -0.2448499724
C 2.153949449, 1.2042287509, 0.0110076866
H 0.237608553, 2.1507311756, -0.2441060395
C 2.8604464353, -0.0003715493, 0.1043560799
H 2.6851149854, -2.1455546163, 0.0826305779
H 2.6861739667, 2.1448950898, 0.0833766671
C 4.3544388239, -0.0008302822, 0.2730320581
H 4.6938134319, -0.8870882764, 0.8104747582
H 4.8427370249, -0.0023342271, -0.7063072264
H 4.694660502, 0.8864313957, 0.8082568397

4-MePhSO$_2$Cl-Cl$^-$, ([(4-Me)-1-Cl]$^-$ (RC)) (MeCN)

S -1.6598108694, -0.0970265608, 0.0000044719
Cl -2.3251048492, -2.1397965539, 0.0000073084
Cl 0.1348632166, 3.7056228734, -0.000022444
O -2.1170631163, 0.44191129, -1.257885455
O -2.1170607004, 0.4419139624, 1.2578941588
C 0.091650939, -0.299058272, 0.000003079
C 0.7593137346, -0.3770986218, -1.2176010809
C 0.7593151536, -0.3770999903, 1.2176060032
C 2.1378297676, -0.5204708854, -1.2043424525
H 0.2147131787, -0.3164653344, -2.1496796269
C 2.1378315358, -0.5204722586, 1.2043455501
H 0.2147159053, -0.316467919, 2.1496854047
C 2.8470402899, -0.5925578546, 0.0000011955
H 2.6719558454, -0.5759824862, -2.1450968269
H 2.6719584961, -0.5759848809, 2.1450993148
C 4.3457821289, -0.7133208426, -0.0000016289
H 4.7027692034, -1.2385953086, -0.886695852
H 4.8022959956, 0.2812249437, -0.0000401253
H 4.7027791449, -1.2385333004, 0.8867250061

4-MePhSO$_2$Cl$_2$$^-$, ([(4-Me)-1-Cl]$^-$ (TS)) (MeCN)

S -1.5372034415, -0.0062477433, -0.0020130678
Cl -1.3601855901, -2.6009151556, -0.0029901181
Cl -1.383379318, 2.5899940022, -0.0008476107
O -2.1793180421, -0.0086852011, -1.2888624767
O -2.1811482617, -0.0097561063, 1.2839190324
C 0.2298280675, 0.0010945889, -0.0007458261
C 0.8934613187, 0.0069029194, -1.2190787749
C 0.891705061, 0.0057112438, 1.218501729
C 2.2798544458, 0.015739628, -1.2031356272
H 0.3442050644, 0.0075587099, -2.1501260772
C 2.2781647861, 0.0145647015, 1.2045497782
H 0.3411339088, 0.0054565795, 2.1487723546

C 2.992938303, 0.017288988, 0.0012360693
H 2.8167578784, 0.0238569675, -2.1440642616
H 2.8137024578, 0.0217554776, 2.1462575083
C 4.4969841283, -0.0049146595, 0.0020981569
H 4.9012730548, 0.480742962, 0.8911991863
H 4.8605156777, -1.0371168867, -0.0032502541
H 4.9022925008, 0.4900399844, -0.8814347206

2, 6-Me$_2$PhSO$_2$Cl ((2, 6-Me$_2$)-1) (gas)
S 1.2895360244, -0.1392514179, -0.5173126923
O 1.6818709877, -1.4752730364, -0.8775580824
O 1.7413072461, 0.981406413, -1.2987535382
Cl 2.0633339408, 0.1730706676, 1.457024738
C -0.4736646979, -0.0240172925, -0.1866577743
C -1.0442600771, 1.2642120915, -0.0990146884
C -1.2212075039, -1.2069852972, -0.0063408919
C -2.4214402209, 1.3336351179, 0.1056881819
C -2.5950446989, -1.0584491155, 0.1908883334
C -3.192707989, 0.1899507323, 0.2354647326
H -2.8879841149, 2.3088045165, 0.1728644493
H -3.1980267123, -1.9483833742, 0.3223894053
H -4.261768121, 0.2727252671, 0.3911562914
C -0.6636229717, -2.6096826445, 0.0137045171
H -0.2924891849, -2.9100538902, -0.965812759
H 0.172283252, -2.703915447, 0.7062069483
H -1.4462590157, -3.3042094728, 0.3175788526
C -0.2807417823, 2.5634689119, -0.1825504727
H 0.6088077584, 2.5567062215, 0.4469505251
H 0.055560044, 2.7618258571, -1.2000401973
H -0.9240161628, 3.3821561918, 0.1388261216

2, 6-Me$_2$PhSO$_2$Cl-Cl$^-$, ([(2, 6-Me$_2$)-1-Cl]$^-$(RC)) (gas)
S -1.3001970499, 0.3605263455, 0.0056037144
Cl -2.1624888198, -1.5309268378, -0.9117434947
Cl 0.5757668204, 2.5432285214, 1.2275663326
O -1.6500866011, 1.3299719233, -0.9930944871
O -1.9019642797, 0.3343177864, 1.3080295186
C 0.4044730679, -0.2386774026, -0.0704589309
C 1.2239961032, 0.162251062, -1.1404370416
C 0.8081479189, -1.1790103197, 0.8928810642
C 2.4791317951, -0.4381229934, -1.2300141278
C 2.0796052805, -1.7313717774, 0.7462842564
C 2.9081444293, -1.377512979, -0.3061379725
H 3.1373973334, -0.1335646673, -2.0354641533
H 2.4182245543, -2.450619735, 1.4832929268

H 3.8923745376, -1.8229661529, -0.3986346447
C 0.8855703175, 1.2506335653, -2.1221330699
H 0.0143631664, 1.0064210676, -2.7297630756
H 0.6625293027, 2.1660727026, -1.569401669
H 1.7382764146, 1.4239391174, -2.7807019465
C -0.0154610478, -1.5982270451, 2.0824010774
H -0.2003510557, -0.7431384366, 2.7336015265
H -0.9872235994, -1.9936951557, 1.7858778205
H 0.5190494116, -2.3663675891, 2.6435733762

2, 6-$Me_2PhSO_2Cl_2^-$, ([(2, 6-Me_2)-1-Cl]$^-$(TS)) (gas)
S 0., 0., 1.297650759
Cl 0.1070296327, 2.5131347908, 0.9992836887
Cl -0.1070296327, -2.5131347908, 0.9992836887
O 1.273503638, -0.0472933008, 1.9656856984
O -1.273503638, 0.0472933008, 1.9656856984
C 0., 0., -0.525015857
C 1.2160371764, -0.2089310732, -1.1911386228
C -1.2160371764, 0.2089310732, -1.1911386228
C 1.1805865661, -0.1984853886, -2.5857978446
C -1.1805865661, 0.1984853886, -2.5857978446
C 0., 0., -3.2834777323
H 2.1071037696, -0.3590802789, -3.1254802741
H -2.1071037696, 0.3590802789, -3.1254802741
H 0., 0., -4.3677856509
C 2.5351628944, -0.4646123106, -0.5115917754
H 2.8341662493, 0.3843367616, 0.1034430737
H 2.4653313942, -1.3313308886, 0.1466824103
H 3.3038729889, -0.6473306406, -1.2644469694
C -2.5351628944, 0.4646123106, -0.5115917754
H -2.8341662493, -0.3843367616, 0.1034430737
H -2.4653313942, 1.3313308886, 0.1466824103
H -3.3038729889, 0.6473306406, -1.2644469694

2, 6-Me_2PhSO_2Cl ((2, 6-Me_2)-1) (MeCN)
S 1.282920702, -0.1380550449, -0.4997074915
O 1.7125947236, -1.4809417703, -0.7975796812
O 1.735637501, 0.9480726308, -1.3340773444
Cl 2.0903291547, 0.2768961272, 1.453146852
C -0.4734718105, -0.0287468323, -0.1769699461
C -1.0406759316, 1.26304072, -0.089559516
C -1.2222458721, -1.2142678825, -0.0052344793
C -2.4167590892, 1.3339317196, 0.1174559875
C -2.5953158199, -1.0609107943, 0.192721193
C -3.189074493, 0.1897379756, 0.2454529105

H -2.8822977758, 2.3089830793, 0.1861146686
H -3.201851215, -1.9489857627, 0.3166803306
H -4.2573103429, 0.2737287537, 0.4033786361
C -0.6756500487, -2.6207351871, -0.0068466095
H -0.2916766409, -2.9002978024, -0.9877996218
H 0.1431843508, -2.7393251495, 0.7019585237
H -1.470182287, -3.3144768866, 0.2635382421
C -0.2783670738, 2.5618145692, -0.1847037954
H 0.6188393079, 2.5600859567, 0.4336509061
H 0.0386833169, 2.7578926905, -1.2090824313
H -0.9178446565, 3.3802998901, 0.1421646664

2, 6-Me_2PhSO_2Cl-Cl^-, ([(2, 6-Me_2)-1-Cl]$^-$(RC)) (MeCN)
S -1.3128737752, 0.3236431102, 0.0090080588
Cl -2.3977978615, -1.333782791, -0.8599953439
Cl 1.1512867103, 3.1380364084, 1.6125108522
O -1.5666677193, 1.4277472061, -0.880613846
O -1.7756026967, 0.3914708852, 1.3726933073
C 0.3632232023, -0.2901773973, -0.0984747638
C 1.1930519472, 0.145801755, -1.1539478057
C 0.7832160061, -1.2139762258, 0.8839917481
C 2.5039712033, -0.3322464994, -1.1544013924
C 2.1050757345, -1.649557524, 0.814458097
C 2.9606096665, -1.2081624138, -0.1829110515
H 3.1723026598, -0.0081004269, -1.9420417274
H 2.4598025486, -2.3538531612, 1.5563974384
H 3.9844509349, -1.5605298055, -0.2118553917
C 0.7943987128, 1.0763838061, -2.2719023733
H -0.1005656341, 0.732490687, -2.7893921137
H 0.5834562834, 2.0779993212, -1.8973472963
H 1.6078051574, 1.1406214877, -2.9930254303
C -0.081436391, -1.7738490904, 1.9865078513
H -0.2771040958, -1.0231096402, 2.7523429133
H -1.0484318158, -2.116079811, 1.6186543087
H 0.4271072225, -2.6176088804, 2.4504709607

2, 6-$Me_2PhSO_2Cl_2^-$, ([(2, 6-Me_2)-1-Cl]$^-$(TS)) (MeCN)
S 0., 0., 1.2677598096
Cl 0.0667647567, 2.6092704738, 1.0765215152
Cl -0.0667647567, -2.6092704738, 1.0765215152
O 1.2680764701, -0.025214347, 1.9426240432
O -1.2680764701, 0.025214347, 1.9426240432
C 0., 0., -0.5293468987
C 1.2238530665, -0.2161161234, -1.1898340179
C -1.2238530665, 0.2161161234, -1.1898340179

C 1.1830670922, -0.2066715185, -2.5840207855
C -1.1830670922, 0.2066715185, -2.5840207855
C 0., 0., -3.2766934235
H 2.1045771368, -0.3717860226, -3.1281428793
H -2.1045771368, 0.3717860226, -3.1281428793
H 0., 0., -4.3597471152
C 2.5479315671, -0.4733840618, -0.5202562323
H 2.8684305168, 0.3826639953, 0.0740370802
H 2.4870585243, -1.3289578043, 0.1527172344
H 3.3046097684, -0.6739813422, -1.2770847677
C -2.5479315671, 0.4733840618, -0.5202562323
H -2.8684305168, -0.3826639953, 0.0740370802
H -2.4870585243, 1.3289578043, 0.1527172344
H -3.3046097684, 0.6739813422, -1.2770847677

2, 4, 6-Me_3PhSO_2Cl ((2, 4, 6-Me_3)-1) (gas)

S -1.6689209247, -0.0036845862, -0.4785012563
O -2.1687459968, 1.2871368111, -0.8704797679
O -2.0719471244, -1.1834367652, -1.1978788607
Cl -2.3540294453, -0.29138293, 1.5358471595
C 0.1031561083, 0.0155102179, -0.2169009688
C 0.7692942116, -1.2228431351, -0.0923791444
C 0.7801064766, 1.2487169688, -0.1162355267
C 2.1519107652, -1.1882361985, 0.0619937494
C 2.1651201401, 1.1981142833, 0.0340285119
C 2.8699000714, 0.0024418461, 0.1104995102
H 2.6816215953, -2.1293061881, 0.1582755331
H 2.7063256467, 2.1346566042, 0.1057862162
C 4.3695705678, -0.0060124593, 0.2359135786
H 4.716258876, -0.8626052048, 0.8164854614
H 4.8334021481, -0.0710339537, -0.753161088
H 4.7366195956, 0.9045138013, 0.7118918707
C 0.0958193675, -2.5735906411, -0.0851979905
H -0.7662003177, -2.5959105021, 0.5812625258
H -0.2672213768, -2.8410827308, -1.0773310677
H 0.806103156, -3.3315949905, 0.2438536301
C 0.1334759559, 2.612703472, -0.1347978929
H -0.2960528562, 2.8412865911, -1.1098525607
H -0.6785456304, 2.6858453773, 0.588034514
H 0.8800859901, 3.3697933125, 0.1032148637

2, 4, 6-Me_3PhSO_2Cl-Cl^-, ([(2, 4, 6-Me_3)-1-Cl]$^-$(RC)) (gas)

S -1.6517040321, 0.0123559517, -0.0362927925
Cl -1.9651779964, -2.0516483592, -0.9199399598
Cl -0.4256797058, 2.7187056493, 1.1858194387

O -2.2147419263, 0.840863378, -1.0636865073
O -2.2555470116, -0.1361203612, 1.2569097441
C 0.1457563123, -0.0943473089, -0.0476477445
C 0.869721488, 0.5217988123, -1.0837288998
C 0.7599666789, -0.8832499693, 0.938776015
C 2.2453589139, 0.3070469382, -1.1037056276
C 2.1411495154, -1.0447883349, 0.8558924749
C 2.9005793398, -0.4654431651, -0.1522025416
H 2.8247351228, 0.7892553627, -1.8840544138
H 2.6337649328, -1.6432586406, 1.615220321
C 0.2848020544, 1.4619600553, -2.1013608243
H -0.4608541749, 0.9791027267, -2.7327423946
H -0.2021731624, 2.2868424345, -1.5757193999
H 1.0821432895, 1.8564931279, -2.7332201051
C 0.0400047993, -1.5337776457, 2.091403367
H -0.3927150216, -0.7734235039, 2.7425585111
H -0.777362102, -2.1717886524, 1.7546068447
H 0.7425943768, -2.1390301909, 2.6663395343
C 4.399013775, -0.6247718863, -0.1859557297
H 4.7748266762, -0.647691545, -1.2117398293
H 4.8878801507, 0.2123605784, 0.3231755092
H 4.7150197075, -1.5435024519, 0.3134100095

2, 4, 6-$Me_3PhSO_2Cl_2^-$, ([(2, 4, 6-Me_3)-1-Cl]$^-$(TS)) (gas)
S 1.599648463, 0.0078810586, -0.0059761984
Cl 1.2950609812, 2.1390810598, -1.3524903493
Cl 1.3306964114, -2.127735987, 1.3466762758
O 2.2651572132, -0.6647432517, -1.0897172636
O 2.2676925635, 0.6866141038, 1.0723672319
C -0.2181175975, -0.0030138519, 0.0017274003
C -0.8882487001, -0.8050816294, -0.9317691964
C -0.8896558829, 0.7889717881, 0.9427491876
C -2.2818376378, -0.7836471026, -0.8988000016
C -2.2831055177, 0.7532873828, 0.9199853999
C -2.9984887788, -0.0164308007, 0.0111360412
H -2.8186639463, -1.4024428924, -1.610728443
H -2.82099114, 1.3595356321, 1.6418501307
C -0.2116186649, -1.69802208, -1.9376390654
H 0.3956551948, -1.1188330404, -2.6335162663
H 0.4540314106, -2.4020244424, -1.4369759124
H -0.9649439228, -2.2535273268, -2.4988856507
C -0.2146078604, 1.6804208942, 1.9509732859
H 0.4042596685, 1.1018837052, 2.6371154256
H 0.4396279952, 2.3956199325, 1.4511253566
H -0.9692374552, 2.2234153332, 2.5226054841

C -4.506201442, 0.005626505, -0.0083938455
H -4.9139176518, -0.9424686381, -0.3669539133
H -4.9148644652, 0.1952353841, 0.986955247
H -4.878215238, 0.7942752638, -0.6712643608

2, 4, 6-Me$_3$PhSO$_2$Cl ((2, 4, 6-Me$_3$)-1]) (MeCN)

S -1.6601067247, -0.0032140898, -0.4581542681
O -2.1945436864, 1.2922162134, -0.7963254157
O -2.0773805657, -1.1570970087, -1.217453211
Cl -2.3650992047, -0.3761348269, 1.5485591401
C 0.1034046121, 0.0200632282, -0.2115744294
C 0.7677477643, -1.2222102032, -0.0885359313
C 0.7810354695, 1.2560458955, -0.1168972699
C 2.1489478764, -1.1882369656, 0.0676324869
C 2.165119521, 1.2015911586, 0.0339353043
C 2.8676907975, 0.0036739591, 0.1165662766
H 2.678372533, -2.1289372182, 0.1636575643
H 2.708795324, 2.1365333139, 0.0995571894
C 4.3654051963, -0.0065382408, 0.2468835768
H 4.706436377, -0.8561876779, 0.8402158673
H 4.8272025295, -0.0908301315, -0.7416299027
H 4.7325574272, 0.9123447936, 0.7053118667
C 0.0964626242, -2.5738091977, -0.0937293093
H -0.7701745097, -2.6054496226, 0.5663590616
H -0.2524032258, -2.836233544, -1.0925775577
H 0.8059605199, -3.3313874507, 0.2354233919
C 0.1431454916, 2.6230893359, -0.1553188035
H -0.2977313563, 2.8316243094, -1.1300467396
H -0.6533315264, 2.7203919526, 0.5817442535
H 0.8995937359, 3.3786910174, 0.0507678586

2, 4, 6-Me$_3$PhSO$_2$Cl-Cl$^-$, ([(2, 4, 6-Me$_3$)-1-Cl]$^-$ (RC) (MeCN)

S -1.6564379528, 0.0388622929, -0.0212300283
Cl -2.4810275901, -1.9078406563, -0.5087936271
Cl 0.3092066396, 3.523510382, 0.8731407613
O -2.1065641244, 0.8923471088, -1.0913186669
O -2.1045848021, 0.294452124, 1.3256008498
C 0.0885120681, -0.3057088232, -0.0887487492
C 0.8302530059, 0.0594626945, -1.2332342825
C 0.6717507499, -0.9637430003, 1.0174882279
C 2.1989404541, -0.1982100761, -1.2007674785
C 2.0429746988, -1.1897183747, 0.9680919426
C 2.8259204266, -0.8047617528, -0.1171172784
H 2.7924438074, 0.0833440063, -2.0625396249
H 2.5118154627, -1.6918569089, 1.8062873357

C 0.2771022537, 0.7037666138, -2.4798015747
H -0.5475549374, 0.1310439265, -2.9030505103
H -0.1008427678, 1.7049810571, -2.2730628979
H 1.0658931845, 0.7788422428, -3.2267373792
C -0.0748880036, -1.4510835842, 2.2349775259
H -0.383822663, -0.6174161586, 2.8657842178
H -0.9762107479, -2.0041271907, 1.9714006658
H 0.571117393, -2.1058916207, 2.8178200564
C 4.3126484529, -1.0289342582, -0.1107156977
H 4.7071443734, -1.1192795657, -1.1234846616
H 4.8172350346, -0.1831501522, 0.3663462676
H 4.5766605838, -1.9262723263, 0.4507936064

2, 4, 6-$Me_3PhSO_2Cl_2^-$, ([(2, 4, 6-Me_3)-1-Cl]$^-$(TS)) (MeCN)

S 1.5685482809, 0.0048432987, -0.003588979
Cl 1.392611448, 2.2974701714, -1.3060667938
Cl 1.4229972412, -2.2886393552, 1.301895707
O 2.2405989005, -0.6126345681, -1.1125767506
O 2.2431732696, 0.6285296059, 1.1003190791
C -0.2136592575, -0.0031349006, 0.0022805776
C -0.8813072506, -0.8001747776, -0.9482686819
C -0.8820939189, 0.7856024684, 0.9589799857
C -2.2725264447, -0.7768900754, -0.9095828835
C -2.2733827963, 0.7487308783, 0.930487071
C -2.9871345109, -0.0154229227, 0.0113217925
H -2.8131913247, -1.3858890935, -1.6246295098
H -2.8146027198, 1.3460186568, 1.6549166436
C -0.2154818272, -1.6822933456, -1.9710816353
H 0.3655536095, -1.0977069432, -2.6848962993
H 0.4695778834, -2.3843969169, -1.4951763296
H -0.9730347057, -2.2431755173, -2.5161898922
C -0.2174299051, 1.6689583225, 1.9814525455
H 0.3743266668, 1.0868011112, 2.6884107673
H 0.4574801824, 2.3796857447, 1.5038136584
H -0.9762993473, 2.2203093147, 2.5343819542
C -4.490980327, 0.0024649143, -0.0049007376
H -4.8958276338, -0.9441881673, -0.3654985095
H -4.897450968, 0.2017399106, 0.9875728286
H -4.8522415447, 0.7903441859, -0.6730516087

2, 6-*i*Pr_2PhSO_2Cl ([(2, 6-*i*Pr_2)-1]) (gas)

S, 0, 0.0089279585, -1.4811586261, -0.529345723
O, 0, -1.3025629333, -1.9671761006, -0.9433298671
O, 0, 1.1705869537, -1.7859175398, -1.3592024646
Cl, 0, 0.3809672294, -2.3718510538, 1.3947096144

C, 0, -0.0138521254, 0.2887145643, -0.1186167389
C, 0, 1.2396872563, 0.9453321098, -0.0105957064
C, 0, -1.2563369956, 0.9603300482, 0.0231487552
C, 0, 1.2088702046, 2.3437999081, 0.0545666635
C, 0, -1.1988504498, 2.3601348428, 0.0805021885
C, 0, 0.0095321448, 3.0475317414, 0.0646877455
H, 0, 2.146098684, 2.8879947011, 0.106427246
H, 0, -2.1251639255, 2.9199412788, 0.1496604353
C, 0, 2.6101743053, 0.2671306555, 0.0798457584
C, 0, 3.4057131763, 0.7754812521, 1.2997599034
C, 0, 3.4055436592, 0.4528975887, -1.2255059003
H, 0, 2.4786219222, -0.802285736, 0.2313839316
H, 0, 2.8230683818, 0.6793280426, 2.2228139386
H, 0, 4.3180240606, 0.177612806, 1.4089544143
H, 0, 3.7117653722, 1.822690733, 1.1963548037
H, 0, 2.8623780846, 0.0312694031, -2.0758862041
H, 0, 3.5961741599, 1.5150988089, -1.4234575714
H, 0, 4.3734507059, -0.0568513856, -1.150845362
C, 0, -2.6355910615, 0.304287616, 0.1358430708
C, 0, -3.373547619, 0.3489253601, -1.2162615922
C, 0, -3.4910903598, 0.9417253934, 1.2496348683
H, 0, -2.5087198375, -0.7414765101, 0.4096291895
H, 0, -2.8038080706, -0.1661416546, -1.99466379
H, 0, -4.3499927526, -0.1422087914, -1.1292247943
H, 0, -3.5399892896, 1.3864689047, -1.5328161201
H, 0, -2.9459099839, 0.9879526529, 2.1991563168
H, 0, -3.8278901682, 1.954044645, 0.9988643064
H, 0, -4.3889422207, 0.3317712002, 1.4017076967
H, 0, 0.0166545337, 4.134019141, 0.0961899874

2, 6-*i*Pr$_2$PhSO$_2$Cl-Cl$^-$ ([(2, 6-*i*Pr$_2$)-1-Cl]$^-$(RC)) (gas)
S, 0, -0.1603328518, -1.5082057136, 0.2186783945
Cl, 0, -1.1462644041, -2.1422767873, -1.7300168402
Cl, 0, 1.0067522824, 0.2741327216, 2.7717647019
O, 0, -1.1820552787, -1.8206752429, 1.206455253
O, 0, 1.0921907982, -2.2482625311, 0.1533783404
C, 0, -0.0120944219, 0.260680885, -0.2370360877
C, 0, -1.2179759579, 0.9893334808, -0.3792179176
C, 0, 1.2590322189, 0.7833286522, -0.568550102
C, 0, -1.1158893197, 2.2659725401, -0.9444553458
C, 0, 1.2703239812, 2.0543000458, -1.1591593831
C, 0, 0.1059078287, 2.7856104709, -1.3589285929
H, 0, -2.0123509291, 2.8646041467, -1.0621355376
H, 0, 2.2243096177, 2.4880034319, -1.4400482145
H, 0, 0.1520538114, 3.771910152, -1.8160381268

C, 0, 2.6065677469, 0.1153273463, -0.2960342058
C, 0, 3.6311242086, 1.1025698173, 0.2908940093
C, 0, 3.140000196, -0.5741452708, -1.5652836311
H, 0, 2.4598728476, -0.6322355907, 0.4789059609
H, 0, 3.2060604065, 1.5903622362, 1.1737383747
H, 0, 4.5198228277, 0.5417009502, 0.6076406832
H, 0, 3.9638611653, 1.8611088088, -0.4305724216
H, 0, 2.4337583002, -1.3251114097, -1.9335493184
H, 0, 3.3169241047, 0.1581334529, -2.3657480036
H, 0, 4.0910473856, -1.0783945584, -1.3490191282
C, 0, -2.5786182563, 0.5294037306, 0.1439152457
C, 0, -3.7728477243, 1.0847608346, -0.6503997898
C, 0, -2.6823488347, 0.8963813442, 1.6404079195
H, 0, -2.6465750697, -0.5541805335, 0.0641677457
H, 0, -3.6630169641, 0.8975750665, -1.7255487817
H, 0, -4.6899964023, 0.588739814, -0.309367399
H, 0, -3.9165926301, 2.1620981213, -0.4984321371
H, 0, -1.8284818086, 0.5107593075, 2.206386856
H, 0, -2.7011600599, 1.9879843369, 1.7608822673
H, 0, -3.6083028143, 0.4836249438, 2.0631382125

2, 6-*i*Pr$_2$PhSO$_2$Cl$_2^-$ ([(2, 6-*i*Pr$_2$)-1-Cl]$^-$(TS)) (gas)

S, 0, 0.000666331, -1.4560671251, -0.000198622
Cl, 0, 0.7885544282, -1.1879346849, -2.4507000593
Cl, 0, -0.7874242577, -1.1889593632, 2.4502031932
O, 0, -1.2175652639, -2.1395655672, -0.4190857426
O, 0, 1.2193769908, -2.1388404178, 0.4184745232
C, 0, 0.0000947467, 0.3944065875, -0.0000624488
C, 0, -1.2442913702, 1.0600414904, -0.062565119
C, 0, 1.2440692144, 1.0608037162, 0.062606935
C, 0, -1.1997735371, 2.4608373753, -0.0771527639
C, 0, 1.1986765566, 2.4615663151, 0.0775488112
C, 0, -0.0007680568, 3.1595166255, 0.0002928694
H, 0, -2.1312410907, 3.0131843807, -0.1376300804
H, 0, 2.1297992102, 3.0144815413, 0.1381301772
H, 0, -0.0011080391, 4.2475665692, 0.0004395723
C, 0, 2.6093150043, 0.3802686222, 0.1577990426
C, 0, 2.9614240586, 0.1224392798, 1.6369391326
C, 0, 3.7311836679, 1.1586150188, -0.5506630168
H, 0, 2.5485818014, -0.5785538326, -0.3523758103
H, 0, 2.1807864403, -0.4602183119, 2.1338961415
H, 0, 3.9061381163, -0.4329494129, 1.7074552367
H, 0, 3.0789535329, 1.0737055183, 2.1746040649
H, 0, 3.4492154049, 1.4063732278, -1.5802115407
H, 0, 4.0058067979, 2.0846352414, -0.0283004656

H, 0, 4.6304797246, 0.5311386578, -0.5859314913
C, 0, -2.6091210694, 0.3787010142, -0.1579815249
C, 0, -2.9611514826, 0.1213472376, -1.6372188817
C, 0, -3.7314092173, 1.1560236398, 0.5509428776
H, 0, -2.5477737872, -0.5803287503, 0.3517193631
H, 0, -2.1802209299, -0.4606887221, -2.1344468809
H, 0, -3.9055854677, -0.4344896738, -1.7079534857
H, 0, -3.079177643, 1.0727881645, -2.174465118
H, 0, -3.4495354126, 1.403360488, 1.5806182617
H, 0, -4.006566188, 2.0821809228, 0.0291080507
H, 0, -4.6303522139, 0.5280232273, 0.585898799

2, 6-*i*Pr$_2$PhSO$_2$Cl ([(2, 6-*i*Pr$_2$)-1]) (MeCN)
S 0.0629658316, -1.480029765, -0.5406201984
O -1.1865000554, -2.0463745452, -0.9855650578
O 1.2481358915, -1.7572907047, -1.316173261
Cl 0.4188955146, -2.3449740701, 1.3949501515
C -0.0646827048, 0.2722328203, -0.1573832667
C 1.1381594951, 1.0133029779, -0.0829452368
C -1.3446962894, 0.8612958289, -0.0320399737
C 1.0126925338, 2.4011346832, -0.0815247075
C -1.3830801743, 2.2558613645, -0.0388907527
C -0.2279354226, 3.0173601572, -0.1000840397
H 1.906141826, 3.0100914577, -0.0561339159
H -2.3408703265, 2.7545872518, 0.0157206911
C 2.5439065842, 0.4323227646, 0.0557992155
C 3.2682138446, 1.0293403109, 1.275655686
C 3.3586722251, 0.6321025104, -1.2308338233
H 2.476217964, -0.6345029239, 0.2406906395
H 2.6754837313, 0.9073660606, 2.1845389133
H 4.2193801236, 0.5112097206, 1.4168281842
H 3.4869279528, 2.0912531268, 1.1505695222
H 2.8641912169, 0.163847497, -2.0823309537
H 3.4904303225, 1.6947799158, -1.4487620411
H 4.3492762955, 0.1851686499, -1.1195136611
C -2.6672340298, 0.1198497727, 0.1499439805
C -3.4753042208, 0.1065674359, -1.1570547309
C -3.4937977255, 0.7121927798, 1.3049790075
H -2.458940731, -0.9084858554, 0.4255284314
H -2.9105577582, -0.3619093821, -1.9633737154
H -4.4041939456, -0.451621653, -1.0187681286
H -3.7331675062, 1.124251594, -1.4612124031
H -2.9057514779, 0.7732491678, 2.2229684657
H -3.8775304399, 1.7083829922, 1.0780283718
H -4.3540463341, 0.066235165, 1.493886021

H -0.2942642111, 4.0983478936, -0.1207264138

2, 6-*i*Pr$_2$PhSO$_2$Cl-Cl$^-$, ([(2, 6-*i*Pr$_2$)-1-Cl]$^-$(RC)) (MeCN)

S -0.000015215, 1.4507136925, -0.0001685567
Cl -0.7771043804, 1.2749759527, -2.4916592524
Cl 0.7770778051, 1.275459476, 2.4913551283
O 1.1986938609, 2.1376237836, -0.3980225113
O -1.1987406596, 2.1376694702, 0.3975570595
C 0.0000066029, -0.3675155875, 0.0000009968
C 1.2463235537, -1.0294861649, -0.0605683704
C -1.2462945163, -1.0295049973, 0.0606924129
C 1.1969160871, -2.4233913371, -0.0710582515
C -1.1968535276, -2.4234070036, 0.0714375188
C 0.0000395975, -3.1148997951, 0.0002527628
H 2.1212494017, -2.9795271182, -0.1270559718
H -2.1211733882, -2.9795548227, 0.1275373249
H 0.0000525735, -4.1981660103, 0.0003517754
C -2.6159644801, -0.3627100487, 0.1456755416
C -2.9769831496, -0.1008719699, 1.6190362795
C -3.7276385624, -1.1582134064, -0.5532513873
H -2.5636322604, 0.5905614127, -0.3687820983
H -2.2103116604, 0.4938640149, 2.115607052
H -3.9265036695, 0.4361916289, 1.6830466757
H -3.0827328242, -1.0465063785, 2.1577966919
H -3.448648421, -1.4210996961, -1.5757189775
H -3.9898835026, -2.0750032507, -0.0212059486
H -4.6284267532, -0.5420963248, -0.595061962
C 2.6159778562, -0.362674417, -0.1456721464
C 2.9769951466, -0.1011045577, -1.6190807903
C 3.7276682283, -1.158020039, 0.5534084531
H 2.5636212911, 0.5906924681, 0.3686059473
H 2.210311886, 0.4935216345, -2.1157649462
H 3.9265040132, 0.4359679699, -1.6831890966
H 3.0827671272, -1.0468374931, -2.1576638414
H 3.4486803513, -1.420720545, 1.5759244055
H 3.9899369043, -2.0749037587, 0.0215365328
H 4.6284416841, -0.5418737817, 0.5951065498

2, 6-*i*Pr$_2$PhSO$_2$Cl$_2^-$, ([(2, 6-*i*Pr$_2$)-1-Cl]$^-$(TS)) (MeCN)

S -0.0917726304, -1.4251363936, 0.4988623599
Cl -1.0122003389, -2.3601024865, -1.2244695801
Cl 1.6503616682, 0.710223735, 3.6704052083
O -1.0508059233, -1.5415454993, 1.5710686776
O 1.1678576554, -2.1136786759, 0.6202649917
C 0.0460367174, 0.2822371812, -0.062120286

C -1.1601615779, 1.0174017196, -0.137164753
C 1.3171000431, 0.836307057, -0.3683742053
C -1.0513270587, 2.3695923674, -0.4601425427
C 1.3298358652, 2.1922977387, -0.692644341
C 0.1728304615, 2.9535683049, -0.7258280154
H -1.9470352289, 2.9734823103, -0.5055181339
H 2.2700401234, 2.668971293, -0.9242082529
H 0.228415399, 4.0070581183, -0.9720478441
C 2.63747348, 0.0708559678, -0.4264358368
C 3.8831575789, 0.9540584911, -0.2811230752
C 2.7088660427, -0.7386897638, -1.7351745127
H 2.6625864419, -0.6262039168, 0.4044257727
H 3.829749348, 1.5835310171, 0.6094258445
H 4.7587325174, 0.3088017668, -0.1850715178
H 4.049062138, 1.5945325937, -1.150275304
H 1.8633191845, -1.4186888863, -1.8393250637
H 2.7147058195, -0.0660878409, -2.5970229973
H 3.6254829808, -1.3323905882, -1.7592144835
C -2.5722727587, 0.4810178108, 0.0970960573
C -3.5153150648, 0.8351179668, -1.064905892
C -3.1187409385, 0.9851156747, 1.4426181561
H -2.5550776727, -0.6004450197, 0.1463381417
H -3.1060999839, 0.4977259412, -2.0194049465
H -4.4757849403, 0.3381163425, -0.9115126397
H -3.708278919, 1.9069522884, -1.1354315953
H -2.4693681417, 0.6798793633, 2.2641932824
H -3.1973061418, 2.074857662, 1.4519195498
H -4.1146831453, 0.5720243594, 1.6187817767

2, 4, 6-*i*Pr$_3$PhSO$_2$Cl ([(2, 4, 6-*i*Pr$_3$)-1]) (MeCN)

Cl 3.0963508043, -0.5803693704, 1.3747582914
S 2.213968046, -0.1860623048, -0.5514069689
O 2.4123847031, -1.3929487804, -1.3183655629
O 2.8488354893, 1.0237391331, -1.0147747648
C 0.4873506558, 0.0492563594, -0.1431424439
C -0.3342872998, -1.1007117024, -0.0536104436
C -1.706534552, -0.8855225456, -0.0296491228
H -2.359764948, -1.7468734497, 0.0088777388
C -2.2689156527, 0.3883092043, -0.0422563473
C -1.413180248, 1.4814457335, 0.0067179333
H -1.8443135104, 2.4721959807, 0.0680203818
C -0.0247264337, 1.3597088249, -0.0089698605
C 0.7952449884, 2.6371741721, 0.1586490168
H 1.8155339355, 2.369398438, 0.4120495426
C 0.828470506, 3.4451522122, -1.1479923415

H 1.2467680324, 2.8549087167, -1.9637174551
H 1.4424028267, 4.3399770204, -1.0214038675
H -0.1786343892, 3.7618041936, -1.4311139864
C 0.2763182819, 3.4946468332, 1.326374016
H 0.2017764505, 2.909404543, 2.2452031202
H -0.7014449948, 3.9340369261, 1.1213411671
H 0.9738802963, 4.3166971061, 1.5016318236
C -3.7722710231, 0.5797228397, -0.0523086677
H -3.9614334545, 1.6566516264, -0.0824008277
C -4.4146751584, 0.0271812211, 1.2309031025
H -5.4886181695, 0.227186689, 1.2309233756
H -3.9797412058, 0.4890056803, 2.1198056337
H -4.2747405188, -1.0538850817, 1.3079381619
C -4.4088664905, -0.0438981778, -1.3046952185
H -5.4819872364, 0.1594781845, -1.32241561
H -4.2734443276, -1.12818617, -1.3187793972
H -3.9677056985, 0.3640278207, -2.2166334416
C 0.1548453483, -2.5421999426, 0.0774724728
H 1.2257339752, -2.543853643, 0.2526067533
C -0.4767400989, -3.2311271716, 1.300283161
H -0.0198034076, -4.2141514064, 1.4346661622
H -1.551634127, -3.3807743028, 1.1839909628
H -0.3098709997, -2.6502398631, 2.2097137861
C -0.1093850336, -3.3377890418, -1.2095265176
H 0.2711209747, -4.356619393, -1.1056419782
H 0.3833060305, -2.8723831862, -2.0636435075
H -1.1804563666, -3.3966289259, -1.4182502724

2, 4, 6-*i*Pr$_3$PhSO$_2$Cl-Cl$^-$, ([(2, 4, 6-*i*Pr$_3$)-1-Cl]$^-$(RC)) (MeCN)

Cl -0.5368165279, -1.5068601158, -3.9412460547
S -2.1494345268, -0.3104327112, -0.2618519903
O -2.5419098525, -1.6970652897, -0.2754434493
O -2.7626297928, 0.5915051096, -1.2077603795
C -0.3782214197, -0.0449084009, -0.165767695
C 0.5162993577, -1.1460974268, -0.0772492241
C 1.8712987479, -0.8398057919, -0.1237876645
H 2.5818479724, -1.6504268399, -0.0707789005
C 2.3580016628, 0.4598339618, -0.2393824898
C 1.443049404, 1.5005315669, -0.2760598174
H 1.8136535501, 2.5148837002, -0.3469602376
C 0.0659839208, 1.2952506459, -0.223036489
C -0.8124479823, 2.5463547288, -0.227769059
H -1.8357435671, 2.2806297656, 0.006848626
C -0.3787989866, 3.5497026836, 0.854183886
H -0.3501417278, 3.0774724336, 1.838352367

H -1.0980905281, 4.3709394643, 0.8913851819
H 0.6031300968, 3.9820133276, 0.6542020007
C -0.8214795448, 3.1894069999, -1.6238647001
H -1.1842456769, 2.4846049508, -2.3732032402
H 0.1806025335, 3.5147088048, -1.9133493473
H -1.4763200575, 4.0639642611, -1.6279812578
C 3.8472459716, 0.7313395861, -0.3132212378
H 3.9752656852, 1.8152692911, -0.3855509756
C 4.4660697783, 0.1008836414, -1.5716461495
H 5.5257261642, 0.3576718355, -1.6399678093
H 3.967860411, 0.4558329661, -2.4762584812
H 4.3853516946, -0.9886417734, -1.5466593508
C 4.5708979057, 0.258185839, 0.9578485724
H 5.6301322841, 0.5207326887, 0.9078676376
H 4.5001942667, -0.8262368672, 1.0727041059
H 4.1445545839, 0.7215993377, 1.850149815
C 0.1155699888, -2.6068703553, 0.119601482
H -0.7462698532, -2.8095390709, -0.5078676846
C 1.1930100699, -3.6172804794, -0.2936591777
H 0.7642820327, -4.6207411243, -0.254050168
H 2.0563508055, -3.6092446154, 0.3756787492
H 1.542562233, -3.4404818321, -1.3128061067
C -0.2935002964, -2.8378377284, 1.5867400242
H -0.6358562789, -3.8661066489, 1.7238949966
H -1.098203669, -2.170281779, 1.8948735231
H 0.5607696917, -2.6709794824, 2.2483232087
Cl -2.7953567547, 0.3733347527, 1.6958722711

2, 4, 6-*i*Pr$_3$PhSO$_2$Cl$_2^-$, ([(2, 4, 6-*i*Pr$_3$)-1-Cl]$^-$(TS)) (MeCN)
Cl -1.7132443575, -1.0687189428, -2.7869255905
S -2.1109714105, -0.3439869691, -0.2827586787
O -2.5918339382, -1.6480040923, 0.0830193322
O -2.9806246574, 0.7187186568, -0.7072118161
C -0.3374015376, -0.0256433203, -0.2067183559
C 0.5401262251, -1.1329549789, -0.1315032806
C 1.8972188517, -0.836494515, -0.0738503119
H 2.596437989, -1.6572752579, -0.0078015068
C 2.3926844343, 0.4630667879, -0.108722711
C 1.4827003431, 1.5081460789, -0.1905189779
H 1.8635612622, 2.5195904984, -0.2170416886
C 0.1030414724, 1.3154278506, -0.2305428366
C -0.7869135037, 2.5505496735, -0.32789765
H -1.7357712724, 2.3243920934, 0.1465510581
C -0.2247984705, 3.7714950348, 0.4139412083
H 0.0440581004, 3.5247058098, 1.4431026555

H -0.989759825, 4.55059093, 0.4402774684
H 0.6516513939, 4.1980401217, -0.0781847308
C -1.0494054071, 2.8848751332, -1.807481788
H -1.475572368, 2.0326206383, -2.3368702852
H -0.1166740963, 3.1672851777, -2.3033191428
H -1.7451819927, 3.7238831045, -1.8856373608
C 3.8833067857, 0.7340170998, -0.0597454618
H 4.0165714359, 1.8190367497, -0.1016826141
C 4.6042536236, 0.126335359, -1.2741065581
H 5.665644573, 0.3843471321, -1.2515302603
H 4.1810044281, 0.4973119781, -2.2100329294
H 4.5240807669, -0.9635503521, -1.2753222573
C 4.5015487679, 0.2371213301, 1.2572316714
H 5.5622822852, 0.4956242256, 1.2978672292
H 4.417769116, -0.8487870025, 1.347077976
H 4.0056360299, 0.6870224802, 2.1199890907
C 0.1259571482, -2.6002494561, -0.0754887811
H -0.7991974234, -2.7141960614, -0.6303737807
C 1.134866838, -3.5485556502, -0.7383912157
H 0.6910751932, -4.5442098181, -0.8054757059
H 2.0602500729, -3.6472775529, -0.1670805171
H 1.3876034485, -3.2204950324, -1.7487672092
C -0.1285032059, -3.0113947475, 1.3860758288
H -0.4941999361, -4.0402684869, 1.4291389596
H -0.8665904294, -2.3628825931, 1.8581871532
H 0.7991572523, -2.954192153, 1.9621557471
Cl -2.2016002358, 0.4317470486, 2.2377159346

$C_6F_5SO_2Cl$ (MeCN)

S 2.0221496418, 0.2390233639, -0.4305646847
O 2.4363896073, -0.7156842434, -1.4219197408
O 2.4239472174, 1.6157618028, -0.4973497572
Cl 2.6906032461, -0.4966625879, 1.4251605594
C 0.2552735931, 0.1254797744, -0.2081096261
C -0.5488747294, 1.2572648275, -0.0523520479
C -0.3503762899, -1.1345858014, -0.2021025424
C -1.9231603821, 1.1273471162, 0.0822254617
C -1.7198684595, -1.2649644424, -0.0631615656
C -2.507721827, -0.1298366865, 0.0756457628
F -0.0498580544, 2.4866488735, -0.0307934656
F -2.6841482641, 2.2113389688, 0.2182217938
F -3.8212248054, -0.2494376514, 0.2051572352
F -2.2840597899, -2.4704452594, -0.0577125975
F 0.375174296, -2.2438640546, -0.307705785

$C_6F_5SO_2Cl$-Cl^-, RC (MeCN)

S 2.0395106607, -0.1425924247, 0.0163129871
Cl 2.7747597645, 1.4442703997, -1.1739665444
Cl -0.3677855783, -2.5545799028, 1.9423625892
O 2.4307536059, -1.3554666561, -0.6442766139
O 2.446435644, 0.1497620312, 1.362969248
C 0.2873338085, 0.1246511265, -0.1554828478
C -0.5185227972, -0.7271804064, -0.9112023029
C -0.3083968751, 1.1924429301, 0.5184261341
C -1.8879348434, -0.5256707894, -0.9681768801
C -1.6747235346, 1.394372669, 0.461925748
C -2.4661683249, 0.5297906753, -0.2817853988
F -0.0276431082, -1.7554347456, -1.5898582793
F 0.4212427187, 2.0543308771, 1.2233438459
F -2.654546535, -1.3569998886, -1.6738962798
F -3.7786252766, 0.7177234007, -0.3373836783
F -2.2325113288, 2.4172607041, 1.1090522731

$C_6F_5SO_2Cl_2^-$, TS (MeCN)

S 0., 0., 1.9298470703
Cl -2.4871852115, 0.0171847965, 1.6615071731
Cl 2.4871852115, -0.0171847965, 1.6615071731
O 0.0079578957, 1.2863150802, 2.5684958417
O -0.0079578957, -1.2863150802, 2.5684958417
C 0., 0., 0.1373331044
C 0.0503945429, 1.1991805854, -0.5681675942
C -0.0503945429, -1.1991805854, -0.5681675942
C 0.0501493015, 1.1986157373, -1.9533328953
C -0.0501493015, -1.1986157373, -1.9533328953
C 0., 0., -2.6487321
F -0.1039578512, -2.3762954748, 0.0509401303
F -0.1000858097, -2.3524774578, -2.6204962674
F 0., 0., -3.9785768579
F 0.1000858097, 2.3524774578, -2.6204962674
F 0.1039578512, 2.3762954748, 0.0509401303

Fluorides in MeCN

$PhSO_2F$ (MeCN)

S -0.0821382066, -1.4557056202, 0.
O -0.5436601815, -1.9814871354, 1.2545390967
O -0.5436601815, -1.9814871354, -1.2545390967
C -0.082445795, 0.3066868395, 0.
C -0.0872039086, 0.9765469927, -1.2205491152
C -0.0872039086, 0.9765469927, 1.2205491152
C -0.0934766619, 2.3651327727, -1.2103257523

H -0.092468163, 0.4244773665, -2.1501686735
C -0.0934766619, 2.3651327727, 1.2103257523
H -0.092468163, 0.4244773665, 2.1501686735
C -0.0949936821, 3.0551333886, 0.
H -0.10025639, 2.9067231217, -2.1472616676
H -0.10025639, 2.9067231217, 2.1472616676
H -0.1016963568, 4.1379647362, 0.
F 1.5208616471, -1.7392775785, 0.

$PhSO_2F_2^-$, SI (MeCN)

S -1.368978376, 0.0000000106, 0.0000011377
O -2.031520193, 1.2896489387, -0.0493498792
O -2.0315200361, -1.2896489387, 0.0493530683
C 0.4257556694, -0.0000000475, -0.0000000038
C 1.093635746, 1.2133572723, -0.0474853668
C 1.093635709, -1.2133574195, 0.0474844851
C 2.4853482345, 1.2057537559, -0.0471699469
H 0.5400820847, 2.1419697742, -0.0838371673
C 2.4853482039, -1.2057540151, 0.0471672729
H 0.5400820242, -2.1419698786, 0.0838370017
C 3.1810411574, -0.0000001594, -0.0000017842
H 3.0239834326, 2.1446513454, -0.0839080105
H 3.0239833719, -2.1446516489, 0.0839046426
H 4.2640908271, -0.0000002012, -0.0000024841
F -1.1719757124, 0.0709205756, 1.8601006877
F -1.1719781432, -0.0709193635, -1.8600986484

$C_6F_5SO_2F$ (MeCN)

S 2.225825272, 0.0998711383, -0.1111566592
O 2.6902160473, -0.76284578, -1.1530849017
O 2.7298373774, 1.423578325, 0.0723508016
C 0.4539908708, 0.0733319235, -0.0383960186
C -0.3106936997, 1.2410482089, -0.0215884519
C -0.2016100954, -1.1603117401, -0.0341058526
C -1.6958741204, 1.170623713, -0.0028039031
C -1.5817207815, -1.2333741785, -0.0107733409
C -2.3295087304, -0.0626374994, 0.0037847674
F 2.5225123016, -0.6843845404, 1.2614989301
F 0.2408471669, 2.4480867352, -0.0313112402
F -2.4194336036, 2.2880564167, 0.0087360059
F -3.6531052243, -0.1259948384, 0.0246696194
F -2.1946364413, -2.4145108918, 0.0009927845
F 0.4914776606, -2.2958719918, -0.0385715406

$C_6F_5SO_2F_2^-$, SI (MeCN)

S 0., 0., 2.1334199516
O -1.2886531736, 0.00721972, 2.788863583
O 1.2886531736, -0.00721972, 2.788863583
C 0., 0., 0.3209819196
C -1.1094053145, 0.4381780878, -0.3901762892
C 1.1094053145, -0.4381780878, -0.3901762892
C -1.1158420989, 0.4341385398, -1.7771719922
C 1.1158420989, -0.4341385398, -1.7771719922
C 0., 0., -2.4740070943
F 0.0149478828, 1.8238087174, 1.900226292
F -0.0149478828, -1.8238087174, 1.900226292
F 2.2092810663, -0.8754537393, 0.2269908697
F 2.1953749006, -0.854341422, -2.4445356058
F 0., 0., -3.8075183466
F -2.1953749006, 0.854341422, -2.4445356058
F -2.2092810663, 0.8754537393, 0.2269908697
